# Supplementary material for: Study protocol for a multi-center stepped-wedge cluster randomized trial to explore the usability and outcomes among young people living with HIV in Kiambu and Kirinyaga counties of Kenya, using an online health portal
Source: PLOS Digit Health. 2025 Jan 17;4(1):e0000380. doi: 10.1371/journal.pdig.0000380 (PMC11741603; doi:10.1371/journal.pdig.0000380)
Supplement: Attachment — Submitted filename: Response to reviewers.docx [file pdig.0000380.s001.docx]

**POINT-BY-POINT RESPONSE**

**REVIEWER 1**

Study Design and Methodology

While the study design as a stepped wedge cluster randomized trial is well-conceived, there needs to be a more detailed explanation of the characteristics and baseline conditions of each cluster. Specifically, please provide clear strategies and methods for minimizing potential heterogeneity between clusters.

*Thank you for the comment.*

*The main strategy that was applied to minimize potential heterogeneity between clusters was stratification. Stratification of clusters was done based on size (small, medium, large), sex (male, female) and viral load suppression to come up with comparable blocks of clusters prior to randomization. This is illustrated in Table 1 below.*

**Table 1: Stratification of clusters based on size, viral load suppression and sex**

| Sub-county | Number of AYPs | Size Strata | *VLS 15-19 | VLS 20-24 | Males (%) |
| --- | --- | --- | --- | --- | --- |
| Thika town | 632 | Large | 80% | 90% | 41% |
| Ruiru | 330 |  | 84% | 89% | 25% |
| Kiambu town | 205 | Medium | 86% | 89% | 32% |
| K Central | 176 |  | 86% | 90% | 55% |
| Gatundu South | 166 |  | 61% | 90% | 40% |
| Juja | 146 |  | 91% | 82% | 35% |
| Kiambaa | 123 |  | 87% | 91% | 27% |
| Kirinyaga South | 116 |  | 91% | 87% | 53% |
| Githunguri | 113 |  | 85% | 89% | 27% |
| Kabete | 110 |  | 94% | 94% | 23% |
| Gatundu North | 107 |  | 92% | 89% | 40% |
| Limuru | 103 |  | 90% | 100% | 35% |
| Kirinyaga West | 88 | Small | 81% | 92% | 37% |
| Kikuyu | 73 |  | 100% | 90% | 16% |
| Kirinyaga East | 58 |  | 100% | 86% | 57% |
| Lari | 51 |  | 100% | 91% | 23% |
| Kirinyaga North | 50 |  | 60% | 68% | 48% |

***VLS –** Viral load suppression

*Clusters within each comparable block (stratum) were then randomly assigned to one of the three sequences for time of crossover from control to intervention using a computer-generated list of random numbers (Table 2).*

**Table 2: Randomization of clusters into sequences**

| **S/No.** | **Cluster** | **15-19 yrs** | **20-24 yrs.** | **Total AYP** | **Strata Size** | **Random**  **number** | **Seq** | **No. of AYP (n=480)** |
| --- | --- | --- | --- | --- | --- | --- | --- | --- |
| 1 | Juja | 48 | 98 | 146 | Medium | 0.91499 | 1 | 186 |
| 2 | Kiambaa | 30 | 80 | 110 | Medium | 0.842108 | 1 |  |
| 3 | Kirinyaga Central | 76 | 83 | 159 | Medium | 0.637954 | 1 |  |
| 4 | Kabete | 19 | 75 | 94 | Small | 0.969301 | 1 |  |
| 5 | Kirinyaga South | 27 | 48 | 75 | Small | 0.832712 | 1 |  |
| 6 | Ruiru | 77 | 253 | 330 | Large | 0.725233 | 1 |  |
| 7 | Gatundu South | 75 | 91 | 166 | Medium | 0.555849 | 2 | 170 |
| 8 | Gatundu North | 52 | 55 | 107 | Medium | 0.546696 | 2 |  |
| 9 | Limuru | 41 | 62 | 103 | Medium | 0.541296 | 2 |  |
| 10 | Lari | 12 | 23 | 35 | Small | 0.701539 | 2 |  |
| 11 | Kirinyaga East | 29 | 26 | 55 | Small | 0.567749 | 2 |  |
| 12 | Thika Level 5 | 172 | 196 | 368 | Large | 0.226127 | 2 |  |
| 13 | Githunguri | 45 | 56 | 101 | Medium | 0.458081 | 3 | 124 |
| 14 | Thika Town | 102 | 135 | 237 | Medium | 0.379402 | 3 |  |
| 15 | Kiambu Township | 87 | 112 | 199 | Medium | 0.062999 | 3 |  |
| 16 | Kirinyaga West | 29 | 42 | 71 | Small | 0.514886 | 3 |  |

**Please see pages 14-15 of the manuscript.*

*Thank you.*

In Figure 3, what does the green color represent in addition to the blue and orange colors? Please add an explanation. Also, there is no period after "B". Please ensure consistency with other sections.

*This table has been adjusted for clarity and consistency as shown below: Green – functions for health care providers, blue – patient health portal components and orange – shared data.*

**Fig 3. Diagrammatic representation of myCareHub™ and functions available to use**

A. **E-Learning Module**

- Disclosure
- Adherence
- Sexual and reproductive health
- Coping skills
- Co-morbidities
- Other topics of interest

B. **Peer to Peer Support**

Patients can act as peer moderators for the forum

D. **Online Patient Info and Diary**

**Patients can report on:**

- mood status
- experience
- screening tools

**Patient can view:**

- current regime
- cd4 count
- viral load

E. **Patient Questionnaire**

- Capture patient’s health beliefs
- Gather real world evidence
- Collect feedback on quality of health care services

G. **Functions for Clinicians & Nurses**

- Track & monitor patient treatment, adherence, and symptoms
- Promote personalized patient care based on visit clinical and self-reported patient response data from the patient diary

F. **Appointment Reminder**

- SMS messages and Reminder notifications

C. **Online Discussion Forum**

Patients can read and post messages to share information and experiences

Patient driven services (A-C)

Data also shared with and used by clinicians and nurses (D-F)

Health care provider functions (G)

**KEY:**

Data Analysis and Statistical Validity

The choice of generalized linear mixed models (GLMM) for data analysis is appropriate, but there is a lack of detailed explanation regarding the model specifications and assumptions. Please elaborate on the variable selection process, interaction terms, and handling of potential confounding variables in the modeling process.

*We have clarified in the manuscript as follows, regarding the model specifications and assumptions:*

Portal Usability Evaluation and Implementation

The evaluation of the portal’s usability and implementation is limited. A detailed description of the tools and methods used for usability assessment, the procedures for collecting and incorporating user feedback, and concrete usage data of the portal’s features should be included. - Especially for the tools used, please specify the original source and whether you obtained approval from the original authors. Additionally, clarify the reliability of these tools.

*Thank you. We acknowledge the omission of critical details relating to portal implementation and have added a dedicated section titled “Study tools” to address these concerns in pages 22 to 26. We have additionally added in pages 28-31 a section on statistical aspects relating to portal-based measurements.*

*A brief point to point response to the key concerns follows:*

Procedures for Collecting and Incorporating User Feedback

Concrete Usage Data of Portal Features

- ***Data Collection:*** *Usage data will be collected through built-in analytics tools within the portal, which track user activity, feature utilization, and frequency of access. This data will provide insights into which features are most and least used.*
-***Analysis:*** *We will analyze the usage data to identify patterns and areas for improvement. Descriptive statistics and usage trends will be reported to highlight key findings related to user behavior and feature performance.*

Ethical Considerations

Although the study mentions ethical approval, there is a lack of detailed explanation regarding participant consent procedures and data privacy protection measures. Given the sensitive nature of health information, please provide clear and specific measures and procedures to ensure data protection and participant privacy

***Thanks for the comment. We have adjusted the manuscript to provide more clarity on our ethical procedures (pages 33-35)***

*Several measures were put in place to ensure data protection and participant privacy.*

*First, all study investigators undertook an online training on the protection of human subjects in research.*

*Second, all study staff that would have access to study data were required to sign and strictly abide by data confidentiality agreements prior to study commencement.*

*Third, towards ensuring participant privacy, interviews were conducted behind closed doors in designated rooms at the participating facilities, while focus group discussions (FGDs) were conducted in designated outdoor spaces within the participating facilities’ premises, away from non-participants.*

*Fourth, participants were recruited into the study only after obtaining written informed consent, and all face-to-face interviews were conducted after obtaining written consent from participants. As part of informed consent procedures, the potential risks and benefits of participating in the study were explained to the participants and it was clarified that the decision to participate would be entirely voluntary. Written consent for audio recordings was also obtained from FGD participants. Participants below the age of 18 years were deemed ‘mature minors’ and were taken through the ‘informed consent’ procedures and given an opportunity to consent on their own behalf. A waiver of parental consent was requested and granted for these adolescents in order to preserve their privacy, considering that they may not have disclosed their HIV status to their parents.*

*Fifth, personal identifiers such as names and addresses were not included on the study data collection tools. Only participants’ initials and a unique study code were documented. Sixth, informed consent & assent forms as well as hard copy data collection tools were stored in restricted access, lockable locations at the enrolling sites. Finally, all electronic data capture and storage tools were password protected, and data was de-identified.*

Results Interpretation and Conclusion Drawing

The interpretation of study results and the drawing of conclusions do not demonstrate the actual impact of portal use on patient activation and secondary outcomes. Please discuss potential confounding factors and limitations in more depth and provide additional analyses and evidence to more convincingly demonstrate the portal’s effectiveness.

*We have included a section on this – pages 35-37, as follows:*

**Results**

This study will assess the impact of myCareHub™ on patient activation, medication adherence, social engagement and viral load suppression among AYP living with HIV. We anticipate improvements in these areas:

1)	Patient activation - we expect that regular interaction with health diaries, reminders, and screening tools through the myCareHub™ platform will lead to increased PAM scores in the intervention group, indicating higher levels of patient engagement and activation in managing their health.

2)	Social engagement - increased use of online communities through the platform is expected to boost SSQ6 social engagement scores, particularly among those who may face barriers to in-person social interactions.

3)	Adherence and viral load suppression - with regular notifications and health management tools, we expect higher medication adherence rates and improved viral suppression among participants who use the portal consistently compared to those in the control group.

**Discussion**

The anticipated results highlight the potential of myCareHub™ to enhance self-management among young people living with HIV. Increased PAM scores would suggest that the portal fosters greater patient activation, while improvements in adherence and viral suppression would confirm its role in supporting clinical outcomes.

Several limitations of the study must be considered. First, selection bias may occur, as participants who are more comfortable with technology might be more likely to engage with the portal, potentially skewing the results. Additionally, confounding factors such as socioeconomic status, access to technology, and health literacy could influence the outcomes. While adjustments will be made during analysis to control for these variables, their impact cannot be entirely eliminated. Another challenge is attrition, as some participants may drop out over time, particularly those with limited access to devices or internet connectivity, which could affect the generalizability of the findings. Technological barriers such as unreliable internet access, compatibility issues with devices, and power outages may also hinder full use of the platform, leading to lower-than-expected engagement and, consequently, a reduced impact on patient outcomes. Finally, the study’s generalizability is limited by its focus on specific counties in Kenya, which may restrict the broader applicability of the findings to other regions or populations.

Please provide the full-term explanations for the abbreviated words within the table.

*All tables have been revised as per recommendations.*

**REVIEWER 2**

The readability of Figure 1 is low, making it difficult to discern the relationship between mechanisms and outcomes.

*Revised as per recommendation:*

There are unnecessarily separated sections. For instance, it would be more appropriate to include the "Justification of the stepped wedge design" in the section where the multicentre stepped wedge cluster randomized trial is first introduced

*Thank you for the comment. Incorporated as follows:*

The intent of the sentence "PAM total scores of 0 and 100 will be considered as missing data, and PAM changes will be analyzed over time and across PAM levels as per the developer’s recommendations" is unclear. It is ambiguous whether it means that missing data will be analyzed over time and across PAM levels as per the developer’s recommendations, or if it separately presents the criteria for missing data and the analysis method for PAM score changes. If it is the latter, additional details on the handling of missing data are required.

*The developer recommends that total scores of 0 and 100 be excluded from analysis. We have clarified this as follows:*

There is a need for adequate explanations of abbreviations included in the tables and figures. For example, "S/No." in Table 4 and "CAC" in Fig 5 are not mentioned in the text. All abbreviations should be fully spelled out at their first occurrence.

*Incorporated in all tables and figures so that they are “self-contained” and can be interpreted separately. Specifically, Table 4 and Fig 5 have been adjusted as follows:*

Abbreviations: CAC – cluster autocorrelation, IAC – individual autocorrelation, PAM – Patient activation measure

**Cluster by Time Variation: High (CAC=0.5)**

Based on definitions in Hooper et al., 2016.

**Cluster by Time Variation: Low (CAC=0.8)**
